# Disulfiram reduces metastatic osteosarcoma tumor burden in an immunocompetent *Balb/c* or-thotopic mouse model

**DOI:** 10.18632/oncotarget.25733

**Published:** 2018-07-10

**Authors:** Jared Anthony Crasto, Mitchell Stephen Fourman, Alejandro Morales-Restrepo, Adel Mahjoub, Jonathan Brendan Mandell, Kavita Ramnath, Jessica C. Tebbets, Rebecca J. Watters, Kurt Richard Weiss

**Affiliations:** ^1^ Musculoskeletal Oncology Laboratory, Department of Orthopaedic Surgery, University of Pittsburgh, Pittsburgh, PA, USA; ^2^ School of Medicine, University of Pittsburgh, Pittsburgh, PA, USA; ^3^ Carnegie Mellon University, Pittsburgh, PA, USA; ^4^ Department of Pharmacology and Chemical Biology, University of Pittsburgh, PA, USA; ^5^ Departments of Anatomic Pathology and General Surgical Oncology, University of Pittsburgh, PA, USA

**Keywords:** osteosarcoma, disulfiram, bad, Akt, aldehyde dehydrogenase

## Abstract

**Introduction:**

The overall survival rate of patients with osteosarcoma (OS) and pulmonary metastases has remained stagnant at 15–30% for several decades. Disulfiram (DSF) is an FDA-approved aldehyde dehydrogenase inhibitor that reduces the metastatic phenotype of OS cells *in vitro*. Here we evaluate its *in vivo* efficacy, as compared to doxorubicin chemotherapy, in a previously-validated orthotopic model of metastatic OS.

**Results:**

All treatment groups displayed a significantly reduced quantitative OS metastatic burden compared with controls. The metastatic burden of Lo DSF-treated animals was equivalent to the DXR group. Ninety-five percent of control animals displayed evidence of metastatic disease, which was significantly greater than all treatment groups.

**Discussion:**

Disulfiram treatment resulted in a reduced burden of OS metastatic disease compared with controls. This was statistically-equivalent to doxorubicin. No additive effect was observed between these two therapies.

**Materials and Methods:**

One-hundred twenty immunocompetent Balb/c mice received proximal tibia paraphyseal injections of 5 × 10^5^ K7M2 murine OS cells. Therapy began three weeks after injection: saline (control), low-dose disulfiram (Lo DSF), high-dose disulfiram (Hi DSF), doxorubicin (DXR), Lo DSF + DXR, and Hi DSF + DXR. Transfemoral amputations were performed at 4 weeks. Quantitative metastatic tumor burden was measured using near-infrared indocyanine green (ICG) angiography.

## INTRODUCTION

Osteosarcoma (OS) is the third most common cancer in the adolescent population and the most common primary malignant bone tumor in both pediatric and adult populations [1–3]. Pulmonary metastatic disease is present in 15–20% of patients at the time of diagnosis. The presence of metastases at diagnosis has been associated with an abysmal (10–15%) five-year survival rate. The poor prognosis of metastatic OS has not improved over the three decades following the advent of modern chemotherapy [4–6].

Aldehyde dehydrogenase (ALDH) is a cancer stem cell (CSC) marker. High ALDH expression has been associated with chemoresistance and CSC survival [7–9]. Our group has demonstrated that OS cells with an aggressive metastatic phenotype express higher levels of ALDH compared to less metastatic cells [10]. Disulfiram (DSF) is a known ALDH inhibitor that has been previously investigated in the treatment of human cancers, acting alone and as a sensitizer to more traditional cytotoxic chemotherapies [11].

Prior *in vitro* studies by our group and others have found that DSF decreases the invasiveness and alters the morphology of OS cells in culture [10, 12, 13]. These studies provide justification for evaluating the activity of DSF *in vivo*. The purpose of the present study was to compare the efficacies of disulfiram (DSF) and doxorubicin (DXR) independently and in combination using a validated, immunocompetent, orthotopic murine model of metastatic OS. Our first hypothesis was that DSF-treated mice would have a lower metastatic disease burden compared with controls. Our second hypothesis was that the molecular effects of DSF on OS cells involves the Bcl-2-associated death promoter (Bad)–Protein Kinase B (Akt) axis.

## RESULTS

### Mortality

The overall mortality rate in this study was 16.7% (20/120, Table 1). Significantly elevated mortality rates compared with saline controls were observed in the high dose (Hi) DSF + DXR (8/20, 40%, *p* = 0.003), DXR (6/20, 30%, *p* = 0.02), and Hi DSF (5/20, 25%, *p* = 0.047) groups. The low dose (Lo) DSF treatment group had a single death, and the Lo DSF + DXR group had no deaths (*p* > 0.99 for both vs. controls). Among the treatment groups, Lo DSF + DXR had significantly reduced mortality compared with DXR alone (*p* = 0.02).

**Table 1:** Mortality data summarized

	Mortality	*p* *value (vs. Saline)*	*p* *value (vs. DXR)*
**Saline**	0 of 20 (0%)	-	-
**DXR**	6 of 20 (30%)	0.0202^*^	-
**Lo DSF**	1 of 20 (5%)	>0.9999	0.0915
**Hi DSF**	5 of 20 (25%)	0.0471^*^	>0.9999
**Lo DSF + DXR**	0 of 20 (0%)	>0.9999	0.0202^*^
**Hi DSF + DXR**	8 of 20 (40%)	0.0033^**^	0.7411

Comparisons made using Fisher's exact tests. ^*^signifies statistical significance (*p* < 0.05^*^, *p* < 0.01^**^).

### Primary and metastatic tumor burden

**Figure 1:**
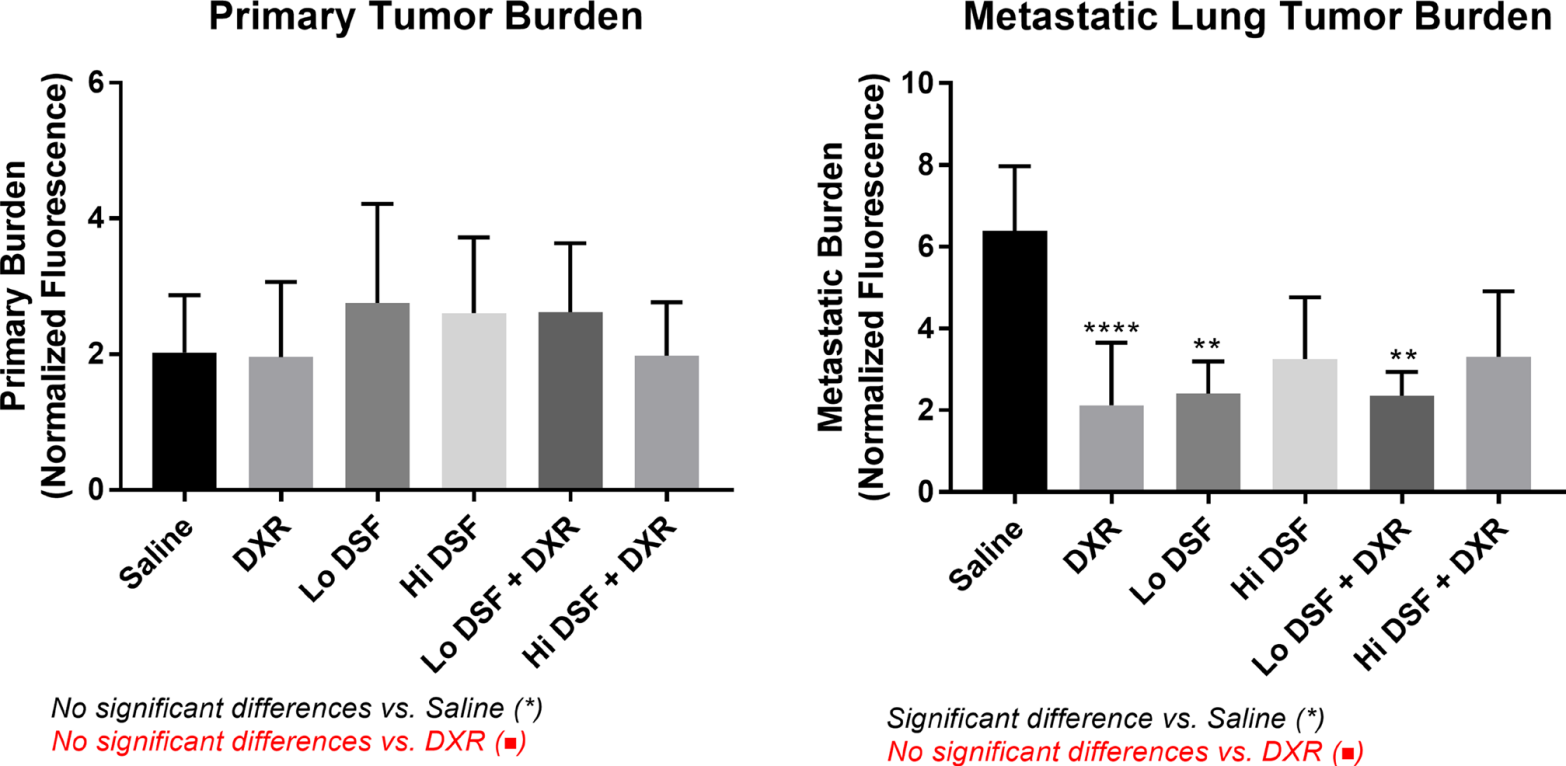
Quantitative Primary (left) and Metastatic (right) Tumor Burden All experimental groups displayed significant (*p* < 0.05) reductions in metastatic tumor burden compared to Saline-treated controls. Error bars depict 95% confidence interval. ^*^signifies significant difference from Saline-treated mice (*p* < 0.01^**^ *p* < 0.0001^****^) on comparison using One-Way ANOVA with Tukey’s post-test. No significant differences existed between DXR and the other treatment groups.

There was a significant difference in primary tumor burden—normalized quantitative indocyanine green (ICG) hindlimb fluorescence [14, 15]—between groups (Figure 1, Table 2). High-resolution ICG measurements were unavailable in 14/120 mice, as noted in Table 2.

**Table 2:** Primary and Metastatic Tumor Burden data summarized

	Primary Tumor Burden (apu)	High-Resolution Measurements	*p* *value (vs. Saline)*	*p* *value (vs. DXR)*	Metastatic Tumor Burden (apu)	High-Resolution Measurements	*p* *value (vs. Saline)*	*p* *value (vs. DXR)*
**Saline**	2.025	19 of 20, 95%	-	-	6.383	20 of 20, 100%	-	-
**DXR**	1.960	18 of 20, 90%	>0.9999	-	2.121	14 of 14, 100%	<0.0001 ^****^	-
**Lo DSF**	2.757	19 of 20, 95%	0.9091	0.8806	2.401	19 of 19, 100%	<0.0001^****^	0.9995
**Hi DSF**	2.607	18 of 20, 90%	0.9665	0.9505	3.256	15 of 15, 100%	0.0041^**^	0.8112
**Lo DSF + DXR**	2.620	18 of 20, 90%	0.9631	0.9461	2.354	20 of 20, 100%	<0.0001^****^	0.9998
**Hi DSF + DXR**	1.977	14 of 20, 70%	>0.9999	>0.9999	3.301	12 of 12, 100%	0.0105^*^	0.8224

Arbitrary perfusion units (apu) normalized against base of tail. Comparisons made using One-Way ANOVA with Tukey’s post-test. ^*^signifies statistical significance (*p* < 0.05^*^, *p* < 0.01^**^, *p* < 0.0001^****^).

Within the surviving population at the 10-week study endpoint, OS metastases were detected in 58.0% (58/100, Table 1). All treatment groups had a significantly decreased metastatic burden compared with controls (*p* < 0.05 for all, Figure 1, Table 2). No other between-group relationships were noted. High-resolution ICG measurements were available in all cases.

### Hindlimb molecular analysis

**Figure 2:**
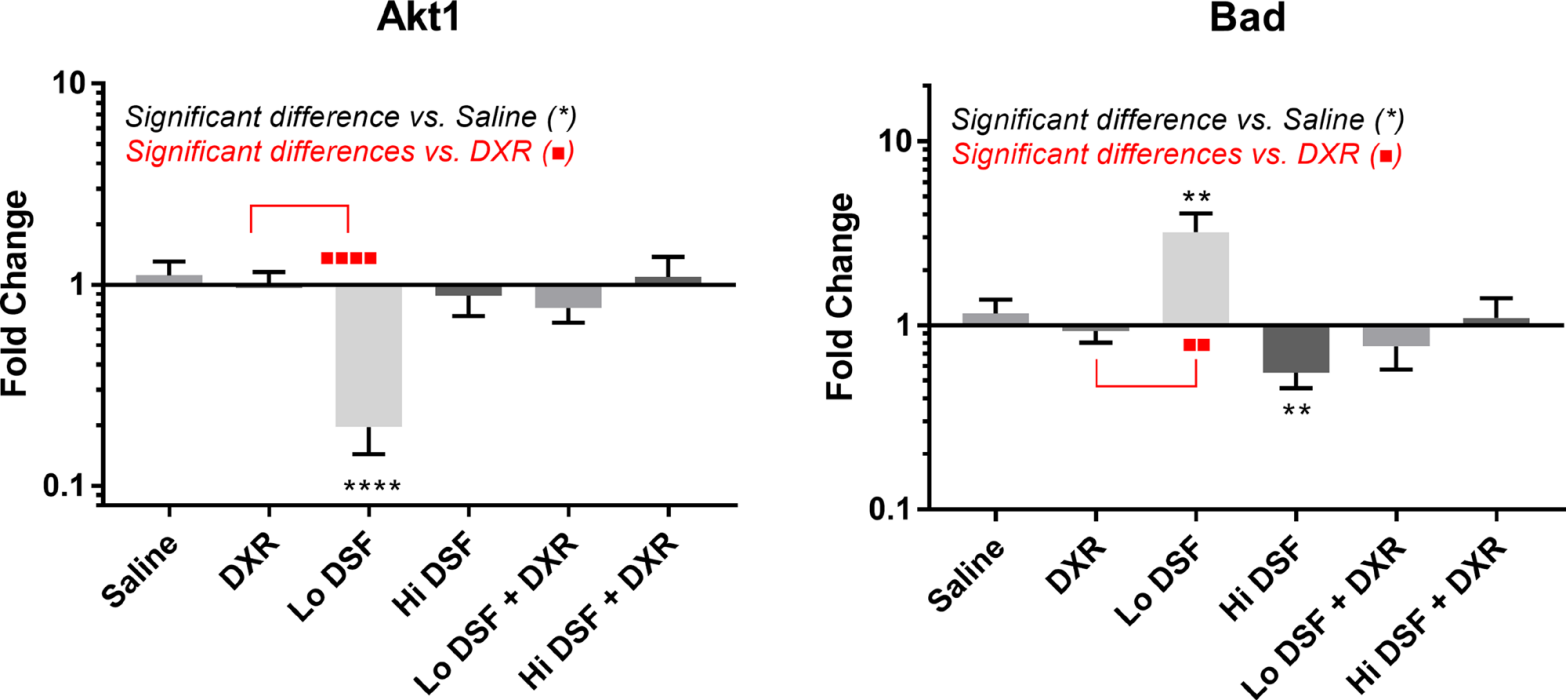
mRNA transcript expression analysis of AKT Serine/Threonine Kinase 1 (Akt, left) and BCL2 Associated Agonist of Cell Death (Bad, right) Fold change is compared to geometric mean of Rps17, Rpl30, and Nono expression levels of saline-treated mice primary tumor samples. Error bars depict 95% confidence interval. ^*^signifies significant difference from Saline-treated mice (*p* < 0.01 ^**^p < 0.0001^****^); ^*^signifies significant difference from Doxorubicin-treated mice (*p* < 0.01^**^, *p* < 0.0001^****^) on comparison using Kruskal–Wallis analysis with Dunn’s multiple comparisons.

**Figure 3:**
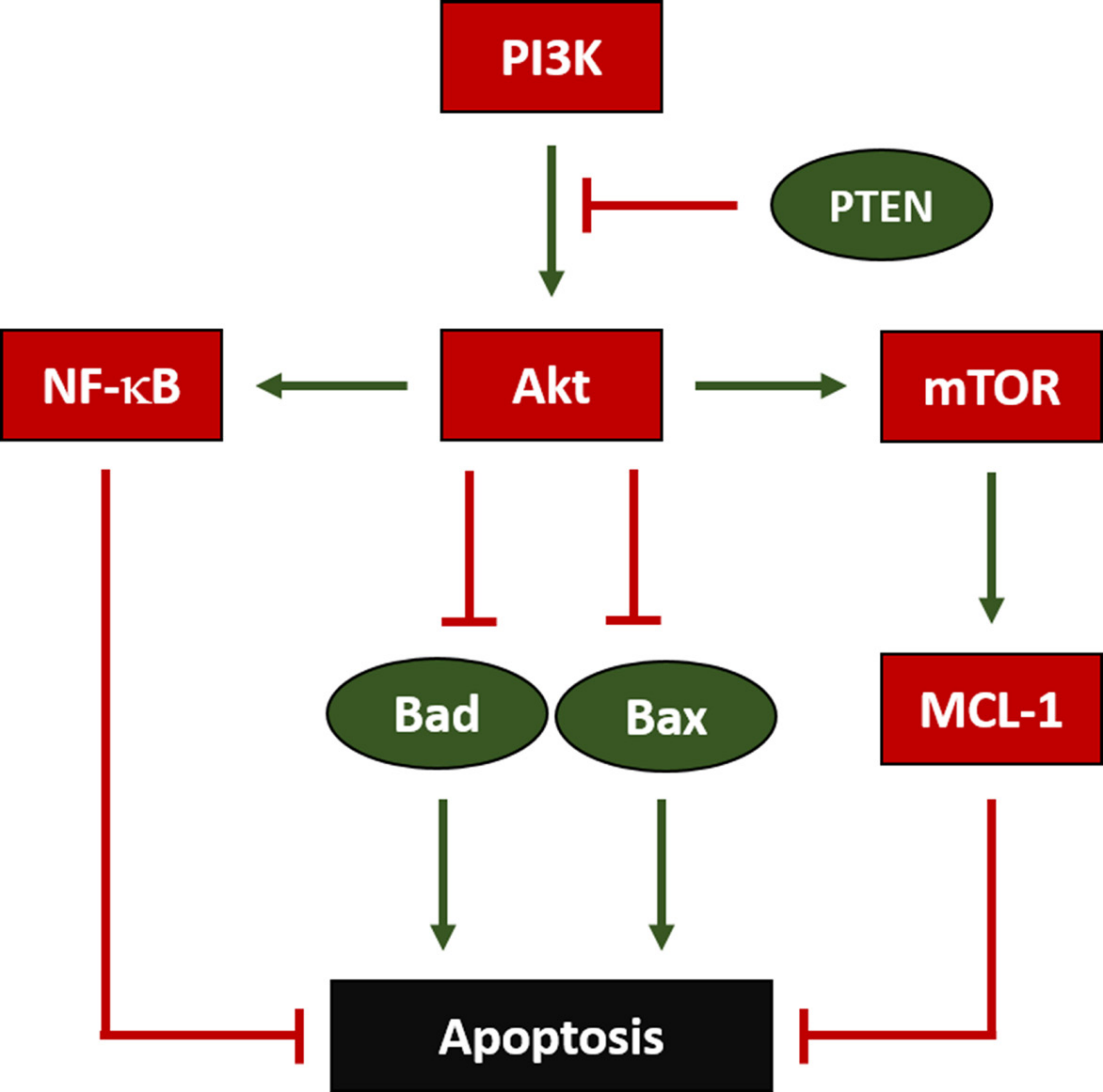
Regulation of Apoptosis in OS Phosphoinositide-3-Kinase (PI3K) enables AKT Serine/Threonine Kinase (Akt) to inhibit BCL2 Associated Agonist of Cell Death (Bad) and BCL2 Associated X Apoptosis Regulator (Bax), which both would otherwise facilitate apoptosis. Additionally, Phosphatase and Tensin Homolog (PTEN) inhibits PI3K’s activation of Akt. Furthermore, Akt also enables Nuclear Factor Kappa B (NF-κB) and Mechanistic Target of Rapamycin (mTOR), which both ultimately block apoptosis; mTOR does so by activating BCL2 Family Apoptosis Regulator (MCL-1).

A complete listing of molecular targets with results can be found in Appendices B and C. A dysregulation of the Bad-Akt axis was noted following Lo DSF treatment, with increased expression of Bad compared with Saline and DXR treated animals (*p* < 0.01 for both) and decreased expression of Akt after Lo DSF treatment vs. Saline and DXR (*p* < 0.0001 for both, Figures 2 and 3). Bad was significantly decreased vs. controls following Hi DSF treatment (*p* = 0.008). Compared with controls, mTOR expression was decreased in all treatment groups except for DXR alone (DXR *p* > 0.99, Lo DSF *p* = 0.003, Hi DSF *p* = 0.003, Lo DSF + DXR *p* < 0.0001, Hi DSF + DXR *p* = 0.046).

No dysregulation of the Notch pathway, including ligand jagged1 (Jag1) and downstream target, transcription factor hairy and enhancer of split-1 (Hes1), was noted in any treatment group vs. controls. Neurogenic locus notch homolog 4 (Notch4) was significantly decreased following Lo DSF treatment vs. Saline (*p* = 0.002).

Bone morphogenetic protein 2 (Bmp2) was significantly decreased following Lo DSF (*p* = 0.02) and Lo DSF + DXR (*p* = 0.0003) treatment vs. Saline. Expression of hypoxia-inducible factor 1-alpha (HIF-1α) was also decreased following Lo DSF treatment vs. controls (*p* = 0.02). Nitric oxide synthase, inducible 2 (NOS2) increased vs. Saline following Lo DSF + DXR treatment (*p* = 0.008). MYC Proto-oncogene, BHLH transcription factor (Myc) expression increased vs. controls following Hi DSF treatment (*p* = 0.0002).

## DISCUSSION

Disulfiram (DSF), an FDA-approved ALDH inhibitor, is also a potent copper chelator with previously described anti-cancer properties. Prior *in vitro* work by our group has shown that DSF can reduce the proliferation and metastatic phenotype of an aggressive mouse OS cell line [13]. Here we have demonstrated in a previously validated OS orthotopic mouse that low-dose DSF has therapeutic equivalence vis-à-vis anti-metastatic effects when compared with doxorubicin (DXR), and may be better tolerated than DXR. Combination therapy with DXR or DSF alone did not result in additional clinical benefit.

Current OS treatment consists of multi-agent neoadjuvant chemotherapy, followed by complete surgical resection, adjuvant therapy, and surveillance for lung metastases [16]. Unfortunately, patients with metastatic disease at the time of diagnosis have a poor response to traditional therapies [1, 2]. Though effective in patients with local disease, treatment with DXR is associated with cardiotoxic side effects in 30% of sarcoma patients [17–19], leading to an estimated fatality rate of 20% [17, 18]. Furthermore, the Childhood Cancer Survivor Study reported a high likelihood of development of second malignancy, coronary artery disease, cerebrovascular disease, renal failure, loss of eyesight, hearing loss, and cognitive dysfunction among adult survivors [20].

Proposed mechanisms for chemotherapy-resistant OS more recently involves cancer stem cells (CSCs) [9]. CSCs are capable of evading traditional chemotherapy, subsequently leading to recurrent or metastatic disease [7–9]. ALDH has been identified as a marker of CSCs [7–9]. Our group has demonstrated that metastatic OS cells have higher levels of ALDH expression and activity, correlating with a metastatic phenotype *in vitro* [10]. Cancer cells with high ALDH have demonstrated enhanced tumorigenicity across multiple subtypes, including lung [21], breast [22], ovarian [23], prostate [24], bladder [25], esophageal [26], melanoma [27], and leukemia [28].

DSF, a derivative of thiuram, has been used in humans to treat alcoholism for more than 60 years [29, 30]. More recent work has pointed to DSF’s potent anti-cancer properties [11]. DSF has been shown to irreversibly inhibit ALDH and chelate copper, thereby promoting apoptosis, inhibiting angiogenesis, and inducing the formation of reactive oxygen species in cancer cells [31]. DSF also slows tumor progression through epigenetic regulation and cellular signaling pathway modulation [11, 32]. As DSF’s side-effect profile is extremely benign in comparison with traditional chemotherapies, it has become an attractive experimental cancer therapy. Recently, a phase I clinical study was published using DSF in combination with chemotherapy to treat glioblastoma multiforme with favorable results and an acceptable safety profile [33]. However, to our knowledge, its efficacy in targeting OS *in vivo* has not been explored. These attributes make DSF an attractive candidate for “drug repurposing” in OS, particularly if doing so could simultaneously target metastatic CSCs more effectively and permit a decrease in the cumulative dose of DXR.

We have shown through a previously validated quantitative methodology [14] that while DSF appears to have minimal effect on primary tumor activity, it may substantially reduce metastatic OS burden. These effects were similar to those of DXR. While not significantly different in this study, DSF also appears to be better tolerated than DXR (5% mortality with DSF vs. 30% with DXR).

The lack of an additive effect between DSF and DXR was surprising, given that a decrease in Akt activity has been associated with an improved response to DXR in breast cancer models [34]. Further, synergy between DXR and targeted mTOR inhibition has been reported in hepatocellular carcinoma *in vivo* models [35]. However, this may be due to DSF and DXR promoting apoptosis via different mechanisms. Apoptosis mediated by DXR has been associated with elevated levels of Akt [36], while DSF was associated with a reduction in Akt in our model. This dissimilar mechanism lies in DXR’s activation of nuclear factor kappa-light-chain-enhancer of activated B cells (NF-κB), which has been found to promote Akt phosphorylation and activation [37]. Its effects on mTOR and HIF-1α suggest that DSF likely operates along the phosphatidylinositol 3-kinase (PI3K)/Akt pathway, further supported by work suggesting that DSF *inhibits* NF-κB activation [38]. It is possible that DSF and DXR operate antagonistically, at least when administered simultaneously as in the model described above. A future area of research will be to explore the effects of temporally-staggered therapy with DSF and DXR.

An additional question raised by our findings lies in the lack of increased potency of DSF when administered at higher doses. The answer may lie in DSF’s mechanism as a copper chelator. As with other metal chelation-dependent chemotherapies, the potency of DSF relies heavily on the availability of copper in the target organism. Multiple prior studies have demonstrated that the efficacy of DSF *in vitro* without available copper is limited [39–41]. While the native copper present in living mice was likely able to permit DSF’s mechanism of action without additional supplementation, it is possible that larger doses of DSF were ineffective due to reduced copper bioavailability. As copper-deficient mice are subject to deleterious effects such as anemia, gut hypoxia, and an immunocompromised state [42, 43], copper deficiency may explain the elevated mortality rates in the Hi DSF and Hi DSF + DXR groups. Future work will focus on studying the impact of copper supplementation and deficiency on the potency of DSF against OS metastasis.

While we have previously validated our methodology for measuring primary tumor and metastatic OS burden utilizing quantitative ICG angiography [14, 15], our model has limitations. First, previous work has noted that the current safe and effective dose of 5mg/kg of ICG used in this study may not be a high enough concentration to detect small micro-metastasis [44]. We chose to keep the dosages consistent with previous studies. Second, our thresholds for establishing a diagnosis of a primary tumor and metastasis were based on the presence of clinically palpable mass and/or ICG signal. Future work should validate the true incidence of metastatic disease based on ICG fluorescence thresholds on a cellular level via histological analysis. Despite this limitation, we believe that our established cut-offs accurately describe the differences observed in this study as the quantitative lung fluorescence measurements do confirm substantially reduced disease burden overall. Lastly, we do not have confirmation that intra-orbital injections as a vehicle do not have systemic side effects on mice, thereby potentially skewing overall study mortality rate. However, this has been discussed in prior work, and no negative consequences of injections up to 150 µL were observed [45]. Finally, a limitation to our molecular analysis lies in the potential “purity” of the assessed samples. While efforts were made to isolate only tumor during primary tumor resection, which was in most cases a discrete and easily dissectible mass, no confirmation was made that samples were completely free from non-tumor cells. While this may slightly skew measurements, we believe that the significant molecular differences observed between DSF and controls are minimally impacted by this potential source of error. Further, the simultaneous evaluation of tumor cells and micro-environmental stroma represents a strength of our study rather than a weakness.

In conclusion, we have found that DSF, when administered as monotherapy at a low dose, is able to reduce the burden of metastatic OS in a previously validated orthotopic mouse model. The effects of low-dose DSF were therapeutically equivalent to DXR in reducing lung metastatic burden. No additive effect was observed between the DSF and DXR combination groups. Molecularly, DSF was found to act along the PI3K-Akt pathway, in line with prior observations. Without question, the role of DSF as an adjuvant to the systemic treatment of OS should be explored in greater depth. Future studies will focus on the optimization of DSF dosing, the utility of copper to potentially augment the efficacy of DSF, and dosing schedules that could potentially maximize the anti-metastatic effects of DSF and decrease the requirement of high doses of DXR.

## MATERIALS AND METHODS

### Mouse and OS cell selection

In an Institutional Animal Care and Use Committee (IACUC) approved study, 120 4–6 week-old female immunocompetent *Balb/c* mice (RRID:IMSR_JAX:000651) received paraphyseal injections of 5 × 10^5^ K7M2 mouse OS cells into their left hindlimb proximal tibias in accordance with a previously published technique [46]. Immune competent *Balb/c* mice were used to better represent the natural history of human OS in light of previous research demonstrating the limitations of immune-deficient mice [47, 48]. K7M2 murine OS cells (RRID:CVCL_V455) were selected because of their pro-metastatic activity and prior characterization in similar orthotopic models [14, 49]. Cells were suspended in phosphobuffered saline in an injectable volume of 20 µL.

### Treatment timeline

Treatments were initiated three weeks after the injection of tumor cells. This timing was selected to mimic the human condition, wherein patients typically present with a painful mass prior to diagnosis and the initiation of neoadjuvant treatment. Saline (20 µL) was injected subcutaneously daily into the dorsal left flank of control mice (*n* = 20). Doxorubicin (DXR, APP- Fresenius Kabi, Lake Zurich, IL, USA) 2 mg/kg per week was diluted in 20 µL of saline and injected retro-orbitally (*n* = 20). Lo DSF of 80 mg/kg per day and Hi DSF of 160 mg/kg per day (*n* = 20, each) (Sigma, St. Louis, MO, USA) were suspended in sesame oil (Sigma, St. Louis, MO, USA) and DMEM, and injected subcutaneously into the dorsal left flank. Combined Lo DSF + DXR (*n* = 20), and Hi DSF + DXR (*n* = 20) treatment groups were evaluated using the same dilution and routes. Transfemoral amputations of the left hindlimbs were performed 4 weeks after OS inoculation, in line with a previously determined natural history of K7M2 tumor growth in immunocompromised SCID/beige mice [47]. Amputations were intended to render the mice free of the primary tumor, such that only micrometastatic disease remained. Hemostasis was achieved, surgical wounds were closed, adjuvant chemotherapy treatments continued without delay. Tumor was harvested, flash frozen, and placed in a −80° C freezer for storage. Euthanasia and lung harvesting was performed 10-weeks after OS cell inoculation.

### Imaging studies

**Figure 4:**
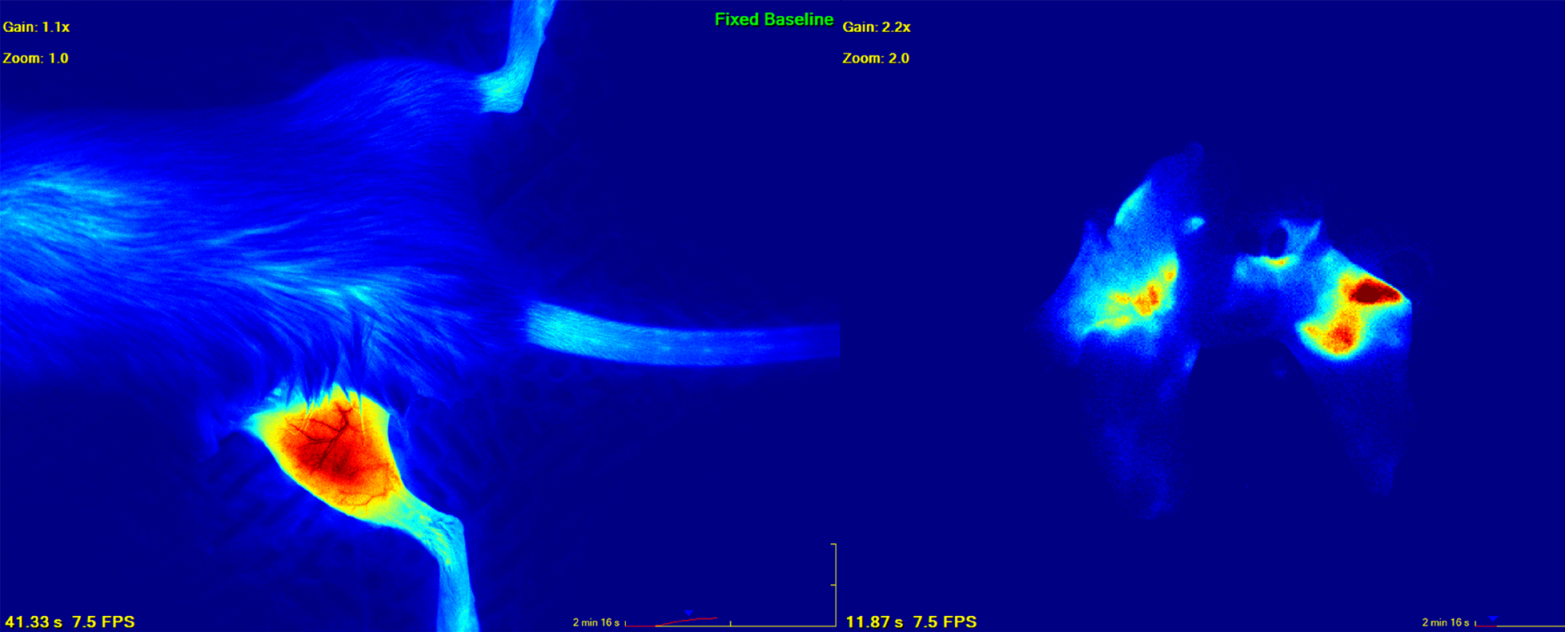
Image depicting primary (left) and metastatic lung (right) osteosarcoma as visualized by indocyanine green dye angiography Quantitative measurements were performed with the SPY-Elite (Novadaq).

Quantitative primary and metastatic tumor burden were evaluated immediately prior to limb amputation at week 4, and immediately following euthanasia and lung harvesting (week 10) with ICG fluorescence angiography SPY Elite, Novadaq, Bonita Springs FL, USA). Imaging utilizing the SPY-Elite device is performed with a 7.5 frame-per-second video acquisition, permitting the measurement of both static and dynamic changes in relative blood flow. Normalization of measured blood flow was against the unaffected base of the mouse’s tail, and measurements were recorded as arbitrary perfusion units (apu). Acquisition and analysis have been previously described [14]. ICG binds tightly to intravascular plasma albumin, and fluoresces upon exposure to 805 nm light, which is within the near-infrared range (Figure 4). Its fluorescence yields an accurate and high-resolution representation of the vasculature of a normal closed system *in vivo*. However, in cases of wounds, vascular injuries, or disorganized tumor vasculature, ICG reliably extravasates when administered at concentrations over 5 mg/kg, remaining within the tumor stroma long after the dye has been cleared from the blood [44].

### Real-time polymerase chain reaction (RT-qPCR) analysis

Total RNA was extracted from primary tumor cells using TRIzol (Thermo Fisher, Waltham, MA, USA), followed by purification using the RNeasy Kit, RNA clean-up protocol (Qiagen, Germantown, MD, USA). In this study, 1 µg of total RNA per sample was used to synthesize cDNA using the High Capacity cDNA Reverse Transcription Kit (Thermo Fisher, Waltham, MA, USA) in a total volume of 20 µL. Amplification of cDNA template samples for the target genes and their primers (Appendix A) were performed with denaturation for 30 min at 98° C followed by 40 cycles of denaturation at 98° C for 10 sec, annealing at 60° C for 30 sec, and extension at 65° C for 5 sec. Values were normalized to the geometric mean of ribosomal protein S17 (*Rps17)*, ribosomal protein L30 (*Rpl30)*, and non-POU domain-containing octamer-binding protein (*Nono)* mRNA. These were selected as housekeeper genes after testing several of our samples on a 30-gene reference panel (Bio-Rad, Hercules, CA, USA). The changes in fluorescence of SYBR green dye in every cycle was monitored and calculated by the Bio-Rad CFX384 system software and the threshold cycle (Ct) for each reaction. The relative amount of PCR products generated from each primer set was determined based on the threshold cycle or Ct value. PCR analysis was performed on each cDNA in triplicate. All primers were supplied by Integrated DNA Technologies (Coralville, IA, USA).

### Statistical analysis

All statistical analyses were performed using Prism 7.0 (GraphPad, La Jolla, CA, USA). Differences in primary tumor growth, tumor metastasis, and mortality in each group were calculated using Fisher’s exact test. Differences in quantitative primary and metastatic tumor burden were compared using one-way analysis of variance with Tukey’s post-test. Differences in mRNA transcript expression between treatment groups for various molecular targets were compared using the Kruskal_Wallis test with Dunn’s multiple comparisons post-test. In all cases, *p* < 0.05 was considered statistically significant.

## SUPPLEMENTARY MATERIALS APPENDICES









## Abbreviations

OS: osteosarcoma
DSF: disulfiram
DXR: doxorubicin
ALDH: aldehyde dehydrogenase
ICG: indocyanine green
