# Supplementary material for: Disulfiram reduces metastatic osteosarcoma tumor burden in an immunocompetent Balb/c or-thotopic mouse model
Source: Oncotarget. 2018 Jul 10;9(53):30163–72. doi: 10.18632/oncotarget.25733 (PMC6059028; doi:10.18632/oncotarget.25733)
Supplement: Supplementary file 1 [file oncotarget-09-30163-s001.pdf]

Disulfiram reduces metastatic osteosarcoma tumor burden in an immunocompetent *Balb/c* or-thotopic mouse model

SUPPLEMENTARY MATERIALS

Supplementary Appendix A: Complete list of target genes and their primers used with cDNA sequences. See Supplementary_Appendix_A

Supplementary Appendix B: Complete list of molecular targets used for PCR analysis and resultant fold change with respect to housekeeper genes ribosomal protein S17 (*Rps17*), ribosomal protein L30 (*Rpl30*), and non-POU domain-containing octamer-binding protein (*Nono*). See Supplementary_Appendix_B

Supplementary Appendix C: Complete list of PCR results for all targets used (Significant results are highlighted in yellow). Fold change values were normalized to the geometric means of housekeeper genes (*Rps17*, *Rpl30,Nono*). See Supplementary_Appendix_C
