# Supplementary material for: Disulfiram reduces metastatic osteosarcoma tumor burden in an immunocompetent Balb/c or-thotopic mouse model
Source: Oncotarget. 2018 Jul 10;9(53):30163–72. doi: 10.18632/oncotarget.25733 (PMC6059028; doi:10.18632/oncotarget.25733)
Supplement: Supplementary file 2 [file oncotarget-09-30163-s002.docx]

| **Gene** | **Primer sequence** | | **Gene** | **Primer sequence** | |
| --- | --- | --- | --- | --- | --- |
| ***Akt1*** | Forward: | GCCCTCAAGTACTCATTCCAG | ***Nos2*** | Forward: | GCAAACATCACATTCAGATCCC |
|  | Reverse: | ACACAATCTCCGCACCATAG |  | Reverse: | TCAGCCTCATGGTAAACACG |
| ***Aldh1a1*** | Forward: | ATCACTGTGTCATCTGCTCTG | ***Notch1*** | Forward: | ATGTCAATGTTCGAGGACCAG |
|  | Reverse: | CCCAGTTCTCTTCCATTTCCAG |  | Reverse: | TCACTGTTGCCTGTCTCAAG |
| ***Bad*** | Forward: | AGGATGAGCGATGAGTTTGAG | ***Notch2*** | Forward: | AAAATCTGCCCTCCACTGG |
|  | Reverse: | CCTTTGCCCAAGTTTCGATC |  | Reverse: | CCGCTTCATAACTTCCCTCTC |
| ***Bax*** | Forward: | TTGGAGATGAACTGGACAGC | ***Notch3*** | Forward: | TGCCAGGGAATTTCAGGTG |
|  | Reverse: | CAGTTGAAGTTGCCATCAGC |  | Reverse: | AGGCAAGAACAGGAAAAGGAG |
| ***Bmp2*** | Forward: | CTCTCAATGGACGTGCCC | ***Notch4*** | Forward: | TGTGAAATCCCTCTAACCTGC |
|  | Reverse: | CAGCTCTGGAATGAGGCC |  | Reverse: | TCTGAGTCTTCCCCTTCTGG |
| ***Hes1*** | Forward: | GGCGAAGGGCAAGAATAAATG | ***Pdgfb*** | Forward: | CCTGCAAGTGTGAGACAGTAG |
|  | Reverse: | GTGCTTCACAGTCATTTCCAG |  | Reverse: | CTTTCGGTGCTTGCCTTTG |
| ***Hif1a*** | Forward: | GAACATCAAGTCAGCAACGTG | ***Pik3r1*** | Forward: | GGATGCTGAATGGTACTGGG |
|  | Reverse: | TTTGACGGATGAGGAATGGG |  | Reverse: | TGTAAGAGTGTAATCGCCGTG |
| ***Jag1*** | Forward: | CTGTCCCACTGGTTTCTCTG | ***Prom1*** | Forward: | AGCAGTACACCAACACCAAG |
|  | Reverse: | GTTCTTGCCCTCATAGTCCTC |  | Reverse: | CGAGGACAGGAGTTACTTTGG |
| ***Mapk3*** | Forward: | ACAAGCGCATCACAGTAGAG | ***Pten*** | Forward: | ACACCGCCAAATTTAACTGC |
|  | Reverse: | AAGATCAACTCCTTCAGCCG |  | Reverse: | GATTGTCATCTTCACTTAGCCATTG |
| ***Mcl1*** | Forward: | TTGTAAGGACGAAACGGGAC | ***Rpl30*** | Forward: | TGGTGTTTGACGCTCTGG |
|  | Reverse: | TCTAGGTCCTGTACGTGGAAG |  | Reverse: | GTTGGAGCCTAGAGTTGATCG |
| ***mTOR*** | Forward: | CAGTTCGCCAGTGGACTGAAG | ***Rps17*** | Forward: | GTTTCCTCTTTTACCGAGACCC |
|  | Reverse: | GCTGGTCATAGAAGCGAGTAGAC |  | Reverse: | GTGGAAGTCATTACCCAGACG |
| ***Myc*** | Forward: | GCTGTTTGAAGGCTGGATTTC | ***Stat3*** | Forward: | GGCACCTTGGATTGAGAGTC |
|  | Reverse: | GATGAAATAGGGCTGTACGGAG |  | Reverse: | CGAAGGTTGTGCTGATAGAGG |
| ***Nfkb1*** | Forward: | AAGACAAGGAGCAGGACATG | ***Vegfa*** | Forward: | GGCAGCTTGAGTTAAACGAAC |
|  | Reverse: | AGCAACATCTTCACATCCCC |  | Reverse: | TGGTGACATGGTTAATCGGTC |
| ***Nono*** | Forward: | GAGAACAAGAGATACGGATGGG |  |  |  |
|  | Reverse: | TCAATCCAAGGGTTCCATCTG |  |  |  |

Appendix A. Complete list of target genes and their primers used with cDNA sequences.
