# Supplementary material for: Disulfiram reduces metastatic osteosarcoma tumor burden in an immunocompetent Balb/c or-thotopic mouse model
Source: Oncotarget. 2018 Jul 10;9(53):30163–72. doi: 10.18632/oncotarget.25733 (PMC6059028; doi:10.18632/oncotarget.25733)
Supplement: Supplementary file 3 [file oncotarget-09-30163-s003.docx]

| Comparison vs. Saline-Treated Mice | | | | | |  | Comparison vs. Doxorubicin-Treated Mice | | | | |
| --- | --- | --- | --- | --- | --- | --- | --- | --- | --- | --- | --- |
|  | **DXR** | **Lo DSF** | **Hi DSF** | **Lo DSF + DXR** | **Hi DSF + DXR** |  |  | **Lo DSF** | **Hi DSF** | **Lo DSF + DXR** | **Hi DSF + DXR** |
| ***Akt*** | - | ⬇ | - | - | - |  | ***Akt*** | ⬇ | - | - | - |
| ***ALDH*** | - | - | - | - | - |  | ***ALDH*** | - | - | - | - |
| ***Bad*** | - | ⬆ | ⬇ | - | - |  | ***Bad*** | ⬆ | - | - | - |
| ***Bax*** | - | - | - | - | - |  | ***Bax*** | - | - | - | - |
| ***Bmp2*** | - | ⬇ | - | ⬇ | - |  | ***Bmp2*** | - | - | - | - |
| ***Hes1*** | - | - | - | - | - |  | ***Hes1*** | - | ⬆ | - | - |
| ***Hif1a*** | - | ⬇ | - | - | - |  | ***Hif1a*** | - | - | - | - |
| ***Jag1*** | - | - | - | - | - |  | ***Jag1*** | - | - | - | - |
| ***Mapk3*** | - | - | - | - | - |  | ***Mapk3*** | - | - | - | - |
| ***MCL-1*** | - | - | - | - | - |  | ***MCL-1*** | - | - | - | - |
| ***mTOR*** | - | ⬇ | ⬇ | ⬇ | ⬇ |  | ***mTOR*** | - | - | - | - |
| ***Myc*** | - | - | ⬇ | - | - |  | ***Myc*** | - | - | - | - |
| ***NF-*k*B*** | - | - | - | - | - |  | ***NF-*k*B*** | - | - | - | - |
| ***Nos2*** | - | - | - | ⬆ | - |  | ***Nos2*** | - | - | - | - |
| ***Notch1*** | - | - | - | - | - |  | ***Notch1*** | - | - | - | - |
| ***Notch2*** | - | - | - | - | - |  | ***Notch2*** | - | - | ⬆ | - |
| ***Notch3*** | - | - | - | - | - |  | ***Notch3*** | - | - | - | - |
| ***Notch4*** | - | ⬇ | - | - | - |  | ***Notch4*** | - | - | - | - |
| ***PDGF-B*** | - | - | - | - | - |  | ***PDGF-B*** | - | - | - | - |
| ***PIK3*** | - | - | - | - | - |  | ***PIK3*** | - | - | - | - |
| ***Prom1*** | - | - | - | - | - |  | ***Prom1*** | - | - | - | - |
| ***PTEN*** | - | - | - | - | - |  | ***PTEN*** | - | - | - | - |
| ***Stat3*** | - | - | - | - | - |  | ***Stat3*** | - | - | - | - |
| ***VEGFA*** | - | - | - | - | - |  | ***VEGFA*** | - | - | - | - |

Appendix B. Complete list of molecular targets used for PCR analysis and resultant fold change with respect to housekeeper genes ribosomal protein S17 (*Rps17)*, ribosomal protein L30 (*Rpl30)*, and non-POU domain-containing octamer-binding protein (*Nono).*
