# Supplementary material for: Disulfiram reduces metastatic osteosarcoma tumor burden in an immunocompetent Balb/c or-thotopic mouse model
Source: Oncotarget. 2018 Jul 10;9(53):30163–72. doi: 10.18632/oncotarget.25733 (PMC6059028; doi:10.18632/oncotarget.25733)
Supplement: Supplementary file 4 [file oncotarget-09-30163-s004.docx]

| **ALDH** | **Fold Change** | ***p value (vs. Saline*** | ***p value (vs. DXR)*** |  | **Hes1** | **Fold Change** | ***p value (vs. Saline*** | ***p value (vs. DXR)*** |
| --- | --- | --- | --- | --- | --- | --- | --- | --- |
| *Saline* | 2.196 | - | - |  | *Saline* | 1.841 | - | - |
| *DXR* | 1.240 | 0.2748 | - |  | *DXR* | 0.576 | 0.3683 | - |
| *Lo DSF* | 0.557 | > 0.9999 | > 0.9999 |  | *Lo DSF* | 0.752 | > 0.9999 | > 0.9999 |
| *Hi DSF* | 6.654 | > 0.9999 | 0.2795 |  | *Hi DSF* | 2.790 | 0.4534 | 0.0009 *** |
| *Lo DSF + DXR* | 1.552 | 0.4781 | > 0.9999 |  | *Lo DSF + DXR* | 1.700 | > 0.9999 | > 0.9999 |
| *Hi DSF + DXR* | 2.221 | > 0.9999 | > 0.9999 |  | *Hi DSF + DXR* | 1.805 | > 0.9999 | 0.2277 |
|  |  |  |  |  |  |  |  |  |
| **Vegfa** | **Fold Change** | ***p value (vs. Saline*** | ***p value (vs. DXR)*** |  | **Notch1** | **Fold Change** | ***p value (vs. Saline*** | ***p value (vs. DXR)*** |
| *Saline* | 1.891 | - | - |  | *Saline* | 1.069 | - | - |
| *DXR* | 0.475 | 0.2502 | - |  | *DXR* | 0.869 | 0.8689 | - |
| *Lo DSF* | 1.046 | > 0.9999 | > 0.9999 |  | *Lo DSF* | 0.905 | > 0.9999 | > 0.9999 |
| *Hi DSF* | 4.127 | > 0.9999 | 0.0741 |  | *Hi DSF* | 1.191 | > 0.9999 | > 0.9999 |
| *Lo DSF + DXR* | 1.324 | > 0.9999 | > 0.9999 |  | *Lo DSF + DXR* | 0.904 | > 0.9999 | > 0.9999 |
| *Hi DSF + DXR* | 1.011 | > 0.9999 | > 0.9999 |  | *Hi DSF + DXR* | 0.922 | > 0.9999 | > 0.9999 |
|  |  |  |  |  |  |  |  |  |
| **mTOR** | **Fold Change** | ***p value (vs. Saline*** | ***p value (vs. DXR)*** |  | **Notch3** | **Fold Change** | ***p value (vs. Saline*** | ***p value (vs. DXR)*** |
| *Saline* | 1.015 | - | - |  | *Saline* | 1.749 | - | - |
| *DXR* | 0.843 | > 0.9999 | - |  | *DXR* | 0.599 | > 0.9999 | - |
| *Lo DSF* | 0.616 | 0.0029 ** | 0.7877 |  | *Lo DSF* | 0.631 | 0.5525 | > 0.9999 |
| *Hi DSF* | 0.695 | 0.0027 ** | > 0.9999 |  | *Hi DSF* | 1.443 | > 0.9999 | > 0.9999 |
| *Lo DSF + DXR* | 0.658 | < 0.0001 **** | 0.0893 |  | *Lo DSF + DXR* | 0.541 | 0.0709 | > 0.9999 |
| *Hi DSF + DXR* | 0.930 | 0.0457 * | > 0.9999 |  | *Hi DSF + DXR* | 0.887 | > 0.9999 | > 0.9999 |
|  |  |  |  |  |  |  |  |  |
| **Myc** | **Fold Change** | ***p value (vs. Saline*** | ***p value (vs. DXR)*** |  | **Jag1** | **Fold Change** | ***p value (vs. Saline*** | ***p value (vs. DXR)*** |
| *Saline* | 1.118 | - | - |  | *Saline* | 1.122 | - | - |
| *DXR* | 0.940 | > 0.9999 | - |  | *DXR* | 0.944 | > 0.9999 | - |
| *Lo DSF* | 0.892 | > 0.9999 | > 0.9999 |  | *Lo DSF* | 0.863 | > 0.9999 | > 0.9999 |
| *Hi DSF* | 0.581 | 0.0002 *** | 0.0938 |  | *Hi DSF* | 1.512 | > 0.9999 | > 0.9999 |
| *Lo DSF + DXR* | 0.820 | 0.0885 | > 0.9999 |  | *Lo DSF + DXR* | 1.007 | > 0.9999 | > 0.9999 |
| *Hi DSF + DXR* | 0.841 | 0.4283 | > 0.9999 |  | *Hi DSF + DXR* | 0.879 | > 0.9999 | > 0.9999 |
|  |  |  |  |  |  |  |  |  |
| **Hif1ɑ** | **Fold Change** | ***p value (vs. Saline*** | ***p value (vs. DXR)*** |  | **Prom1** | **Fold Change** | ***p value (vs. Saline*** | ***p value (vs. DXR)*** |
| *Saline* | 1.067 | - | - |  | *Saline* | 2.551 | - | - |
| *DXR* | 0.784 | > 0.9999 | - |  | *DXR* | 4.989 | 0.5832 | - |
| *Lo DSF* | 0.596 | 0.0176 * | > 0.9999 |  | *Lo DSF* | 1.100 | > 0.9999 | > 0.9999 |
| *Hi DSF* | 0.853 | 0.5077 | > 0.9999 |  | *Hi DSF* | 4.696 | > 0.9999 | 0.4508 |
| *Lo DSF + DXR* | 0.886 | > 0.9999 | > 0.9999 |  | *Lo DSF + DXR* | 1.734 | > 0.9999 | > 0.9999 |
| *Hi DSF + DXR* | 1.153 | > 0.9999 | > 0.9999 |  | *Hi DSF + DXR* | 8.283 | > 0.9999 | > 0.9999 |
|  |  |  |  |  |  |  |  |  |
| **Bmp2** | **Fold Change** | ***p value (vs. Saline*** | ***p value (vs. DXR)*** |  | **Nos2** | **Fold Change** | ***p value (vs. Saline*** | ***p value (vs. DXR)*** |
| *Saline* | 1.119 | - | - |  | *Saline* | 1.098 | - | - |
| *DXR* | 0.643 | 0.1599 | - |  | *DXR* | 1.460 | > 0.9999 | - |
| *Lo DSF* | 0.587 | 0.0169 * | > 0.9999 |  | *Lo DSF* | 1.375 | > 0.9999 | > 0.9999 |
| *Hi DSF* | 1.021 | > 0.9999 | > 0.9999 |  | *Hi DSF* | 2.758 | > 0.9999 | > 0.9999 |
| *Lo DSF + DXR* | 0.593 | 0.0003 *** | > 0.9999 |  | *Lo DSF + DXR* | 8.720 | 0.0080 ** | 0.1481 |
| *Hi DSF + DXR* | 0.766 | 0.7746 | > 0.9999 |  | *Hi DSF + DXR* | 3.990 | 0.1752 | 0.7743 |
|  |  |  |  |  |  |  |  |  |
| **Akt1** | **Fold Change** | ***p value (vs. Saline*** | ***p value (vs. DXR)*** |  | **Notch4** | **Fold Change** | ***p value (vs. Saline*** | ***p value (vs. DXR)*** |
| *Saline* | 1.114 | - | - |  | *Saline* | 1.106 | - | - |
| *DXR* | 1.007 | > 0.9999 | - |  | *DXR* | 1.069 | > 0.9999 | - |
| *Lo DSF* | 0.196 | < 0.0001 **** | < 0.0001 **** |  | *Lo DSF* | 0.495 | 0.0016 ** | 0.1737 |
| *Hi DSF* | 0.883 | > 0.9999 | > 0.9999 |  | *Hi DSF* | 1.330 | > 0.9999 | > 0.9999 |
| *Lo DSF + DXR* | 0.768 | 0.1357 | 0.7709 |  | *Lo DSF + DXR* | 0.894 | 0.5072 | > 0.9999 |
| *Hi DSF + DXR* | 1.091 | > 0.9999 | > 0.9999 |  | *Hi DSF + DXR* | 1.201 | > 0.9999 | > 0.9999 |
|  |  |  |  |  |  |  |  |  |
| **Bad** | **Fold Change** | ***p value (vs. Saline*** | ***p value (vs. DXR)*** |  | **Notch2** | **Fold Change** | ***p value (vs. Saline*** | ***p value (vs. DXR)*** |
| *Saline* | 1.160 | - | - |  | *Saline* | 1.038 | - | - |
| *DXR* | 0.932 | > 0.9999 | - |  | *DXR* | 0.683 | 0.0945 | - |
| *Lo DSF* | 3.205 | 0.0019 ** | 0.0021 ** |  | *Lo DSF* | 0.722 | 0.1120 | > 0.9999 |
| *Hi DSF* | 0.554 | 0.0075 ** | 0.1672 |  | *Hi DSF* | 0.716 | 0.0669 | > 0.9999 |
| *Lo DSF + DXR* | 0.770 | 0.0710 | 0.9473 |  | *Lo DSF + DXR* | 1.383 | > 0.9999 | 0.0035 ** |
| *Hi DSF + DXR* | 1.099 | > 0.9999 | > 0.9999 |  | *Hi DSF + DXR* | 1.126 | > 0.9999 | 0.0972 |

Appendix C. Complete list of PCR results for all targets used (Significant results are highlighted in yellow). Fold change values were normalized to the geometric means of housekeeper genes (*Rps17, Rpl30,Nono)*.
